# Evolution of *E*. *coli* on [U-^13^C]Glucose Reveals a Negligible Isotopic Influence on Metabolism and Physiology

**DOI:** 10.1371/journal.pone.0151130

**Published:** 2016-03-10

**Authors:** Troy E. Sandberg, Christopher P. Long, Jacqueline E. Gonzalez, Adam M. Feist, Maciek R. Antoniewicz, Bernhard O. Palsson

**Affiliations:** 1 Department of Bioengineering, University of California San Diego, La Jolla, CA, 92093, United States of America; 2 Department of Chemical and Biomolecular Engineering, Metabolic Engineering and Systems Biology Laboratory, University of Delaware, Newark, DE, 19716, United States of America; 3 Novo Nordisk Foundation Center for Biosustainability, Technical University of Denmark, 2800, Lyngby, Denmark; University of Houston, UNITED STATES

## Abstract

^13^C-Metabolic flux analysis (^13^C-MFA) traditionally assumes that kinetic isotope effects from isotopically labeled compounds do not appreciably alter cellular growth or metabolism, despite indications that some biochemical reactions can be non-negligibly impacted. Here, populations of *Escherichia coli* were adaptively evolved for ~1000 generations on uniformly labeled ^13^C-glucose, a commonly used isotope for ^13^C-MFA. Phenotypic characterization of these evolved strains revealed ~40% increases in growth rate, with no significant difference in fitness when grown on either labeled (^13^C) or unlabeled (^12^C) glucose. The evolved strains displayed decreased biomass yields, increased glucose and oxygen uptake, and increased acetate production, mimicking what is observed after adaptive evolution on unlabeled glucose. Furthermore, full genome re-sequencing revealed that the key genetic changes underlying these phenotypic alterations were essentially the same as those acquired during adaptive evolution on unlabeled glucose. Additionally, glucose competition experiments demonstrated that the wild-type exhibits no isotopic preference for unlabeled glucose, and the evolved strains have no preference for labeled glucose. Overall, the results of this study indicate that there are no significant differences between ^12^C and ^13^C-glucose as a carbon source for *E*. *coli* growth.

## Introduction

Metabolic flux analysis (MFA) has become an invaluable technique with which to probe the metabolic flux states of an organism [1, 2]. Knowledge of these intracellular fluxes is frequently used in complement with metabolic engineering, where it can be utilized to guide the rational design of genetic manipulations necessary for chemical production [3]. MFA relies on the isotopic labeling of a “tracer” compound that is passed through the reaction network of a cell, and ^13^C is typically used as the stable isotope of choice [4]. Inherent to ^13^C-MFA experiments is the assumption that the labeled compound is not metabolized differently than the unlabeled form, but the altered mass of the isotope can cause measurable kinetic isotope effects for chemical reactions. Evidentially, although ^13^C is only 8% more massive than ^12^C, plants are known to discriminate against the heavier isotope when it comes to carbon fixation [5], and recent work has indicated that neglecting kinetic isotope effects in ^13^C-MFA could potentially result in errors on the same scale as GC-MS measurement errors [6]. Thus, establishing that ^13^C and ^12^C can be treated interchangeably when it comes to metabolic fluxes is important for the continued use of ^13^C-MFA as a robust experimental technique.

Adaptive laboratory evolution (ALE) involves the continuous culturing of microorganisms in a controlled setting such that natural selection for beneficial mutations will lead to cells with improved fitness. This approach can be used for a number of purposes, such as increasing cellular tolerance to some chemical, determining how a metabolic network adapts to engineered alterations, or simply optimizing growth rate on a particular substrate [7]. ALE is ideally suited to investigate the potential metabolic impact of heavier carbon because there have already been studies on evolution to unlabeled glucose [8, 9], and long term culturing serves to amplify any differences between experiments–previously, very minor differences in experimental methodology and starting strain were found to lead to vastly different mutations following ALE experiments [10, 11]. By comparing with an unlabeled glucose evolution and keeping all variables the same except for the exchange of ^13^C with ^12^C, effects of the heavier isotope can be determined. For example, if the stronger C-C bonds caused by ^13^C were indeed impacting cellular metabolism, mutations in C-C bond breaking enzymes could potentially be enriched for. Moreover, isotopically-induced growth differences, if they exist, are more likely to become apparent in the excess nutrient conditions of an ALE experiment where the only limiting factor to growth is how quickly the cells can utilize the nutrients. For these reasons, we sought to investigate the interchangeability of ^12^C and ^13^C glucose in *E*. *coli* metabolism by evolving cultures onto [U-^13^C]glucose. These evolved strains were then characterized phenotypically and genetically.

## Results and Discussion

### Evolved Phenotypic Changes

**Fig 1:**
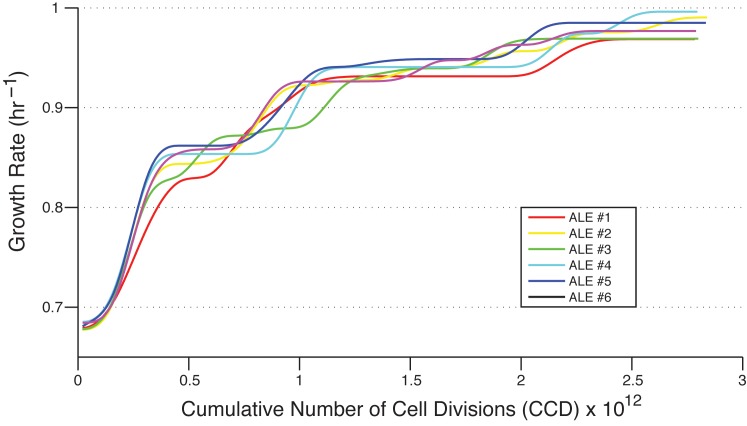
Fitness trajectories. Growth rates of the evolving strains over the course of the ALE as they adapted to growth on ^13^C glucose.

Wild-type *Escherichia coli* K-12 MG1655 was used to establish six independent cultures that were serially propagated in M9 minimal medium with uniformly labeled ^13^C-glucose as the sole carbon source. An automated system was used [10] to perform this Adaptive Laboratory Evolution (ALE) experiment, continually tracking the cultures’ growth rates and passing them to fresh media flasks before depletion of the glucose could serve as a limiting factor to growth. Thus, selection only for cells with faster exponential-phase growth rate was ensured. The fitness trajectories of the evolving cultures very closely resemble those of a previous ALE experiment by LaCroix *et al*. in which the evolution environment was the same except for the use of naturally labeled glucose (referred to as ^12^C-glucose herein) [9]. Noticeable jumps in the population growth rate occurred as fitter mutants emerged and gained dominance in the cultures, but over time these diminished as the trajectories asymptotically approached the “optimal growth rate” (Fig 1). Cultures were evolved for 40 days, corresponding with an average of 963 generations, or 2.82 x 10^12^ cumulative number of cell divisions (CCD) per population [12]. Using CCD as the metric of evolutionary time is more beneficial in an ALE experiment as it factors in the population subsampling that accompanies serial passage of cultures [10]. At the endpoint of the adaptive evolution, the evolved populations were on average 44% fitter (i.e. displayed higher growth rate) than the wild-type ancestor.

**Fig 2:**
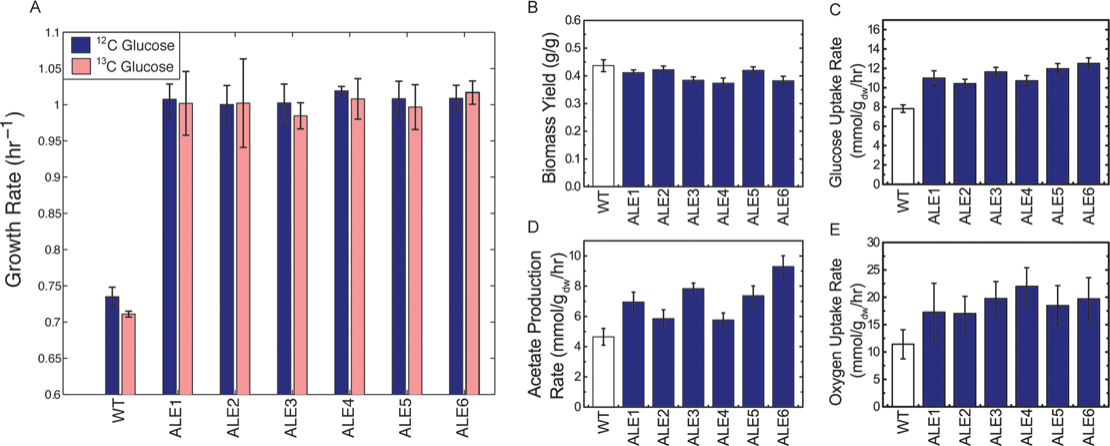
Phenotypic characterization of the wild-type and evolved strains. (A) Growth rates on ^12^C-glucose and ^13^C-glucose. (B) Biomass yields on ^13^C-glucose. (C) Glucose uptake rates on ^13^C-glucose. (D) Acetate production rates on ^13^C-glucose. (E) Oxygen uptake rates on ^13^C-glucose. All error bars represent standard error of the mean (n = 3).

Clones were isolated from the evolved endpoint populations and subjected to phenotypic assays. Despite not having been exposed to ^12^C-glucose over the course of the evolution, the endpoint clones nevertheless demonstrated equivalent fitness on this substrate as they did on ^13^C-glucose (Fig 2A). Although in the wild-type strain there appears to be a slight growth advantage on ^12^C vs. ^13^C-glucose (3 ± 2%), in the evolved strains there is no such statistically significant advantage (paired t-test, *p* = 0.18). Again drawing a close parallel with the evolution of LaCroix *et al*., biomass yields of both ^12^C- [9] and ^13^C-evolved strains slightly decreased, glucose uptake rates increased, and acetate production rates spanned a wide range of increases, indicating that the cells employed different metabolic tactics to realize equivalent fitness improvements (Fig 2B–2D). Unassayed in the LaCroix study, oxygen uptake rates also increased significantly, from 49 to 92% (Fig 2E). Together, these phenotypic data types and evolutionary trajectories, in comparison with equivalent data from evolution onto ^12^C-glucose, do not point to any differences caused by the ^13^C isotope.

Additionally, due to results from the genetic analyses (discussed below), it was decided to run a smaller scale ALE. This “reALE” proceeded for ten days and evolved triplicate cultures of the wild-type used previously, as well as triplicate of a strain from the LaCroix study pre-evolved on ^12^C-glucose. This shorter evolutionary time scale only allowed the wild-type cultures to undergo the first main jump in fitness (Fig 1), while the pre-evolved ^12^C-cultures did not appreciably change in growth rate at all (S1 Fig). Colonies isolated from the endpoints again had essentially identical growth rates on ^12^C and ^13^C-glucose (S2 Fig).

### Evolved Genetic Changes

**Fig 3:**
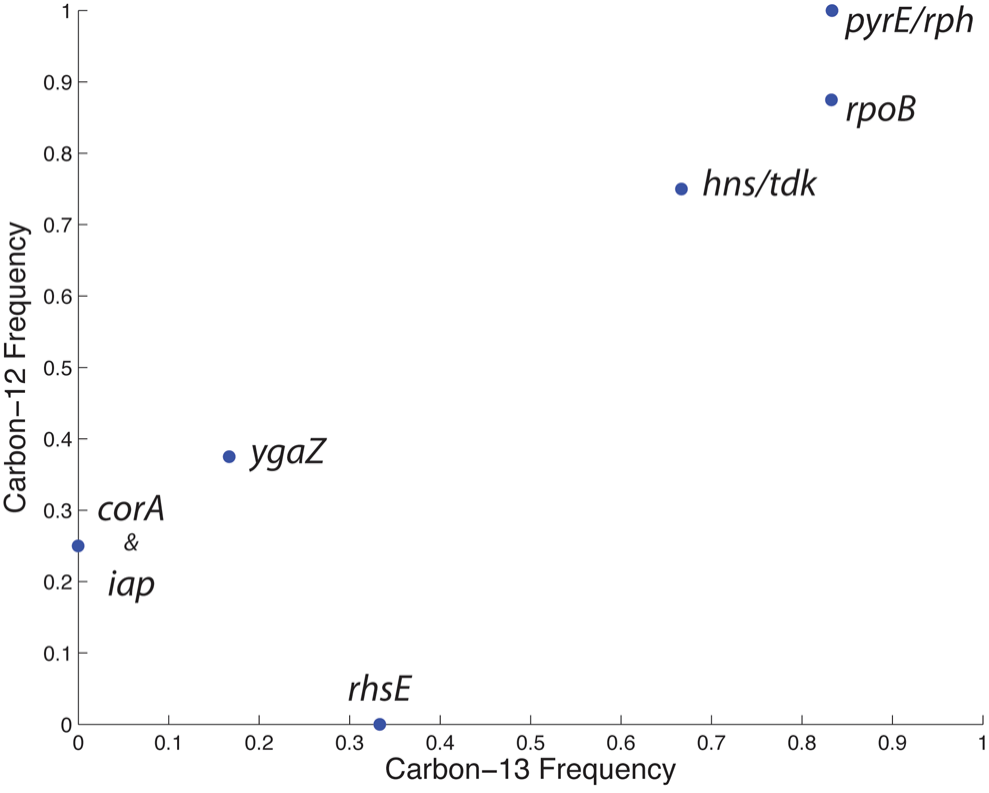
Mutational frequency in evolved endpoint strains. Comparison of the most frequently mutated genes observed in evolved endpoint strains grown on either ^12^C-glucose or ^13^C-glucose.

Although evolution on ^12^C and ^13^C-glucose yields essentially equivalent phenotypes, the genetic adaptations underlying these changes could potentially differ, which would point to isotopic effects. To investigate this possibility, whole genome sequencing was performed on the phenotypically characterized endpoint clones to identify the mutations they had acquired (Table 1). On average, each evolved strain had about 6 mutations, with as many as 11 and as few as 3. Four genes or intergenic regions mutated in parallel across two or more of the strains. By comparing these mutations with those observed in the ^12^C-evolved endpoint strains [9], it was found that alterations to the same three key genetic regions (*pyrE*/*rph*, *rpoB*, and *hns/tdk*) appear to be responsible for the majority of fitness increases (Fig 3). The gene *ygaZ* mutated several times in the ^12^C-evolved strains but only once here, while *corA* and *iap* were not observed to mutate. However, this is unsurprising given that the ^12^C-adapted strains evolved for up to twice as long as this ^13^C evolution (for reasons of ^13^C resource conservation, and because growth rates for the ^13^C ALEs had already visibly leveled off). Had the ^13^C evolution proceeded for longer, it is likely that more of these smaller-effect mutations would have had time to fix in the populations, just as *ygaZ* started to.

**Table 1:** Mutations identified in the endpoints of the ^13^C-evolution.

Mutation^a^	Gene	Protein change	ALE1	ALE2	ALE3	ALE4	ALE5	ALE6
G→T	*murC*	V13L (GTG→TTG)		X				
T→G	*lolB/hemA*	intergenic (-142/-72)						X
IS2	*hns/tdk*	intergenic (-75/-526)						X
IS1	*hns/tdk*	intergenic (-112/-486)			X			
IS1	*hns/tdk*	intergenic (-116/-481)	X					
IS5	*hns/tdk*	intergenic (-258/-344)					X	
G→C	*ycjV*	pseudogene (664/957 nt)						X
T→C	*rhsE*	V429A (GTT→GCT)		X				X
T→C	*rhsE*	F431L (TTT→CTT)		X				X
C→T	*rhsE*	Y432Y (TAC→TAT)		X				X
C→A	*rhsE*	G438G (GGC→GGA)		X				X
C→T	*rhsE*	L443L (CTC→CTT)		X				X
Δ6 bp	*pscG*	coding (778-783/939 nt)		X				
+GGCTGTCA	*yfjR*	coding (32/702 nt)						X
T→G	*ygaY/ygaZ*	intergenic (+59/-65)				X		
+C	*barA*	coding (2425/2757 nt)		X				
T→A	*ptsP*	Q538L (CAG→CTG)				X		
Δ13 bp	*nirC*	coding (414-426/807 nt)		X				
Δ1 bp	*pyrE/rph*	intergenic (-41/+54)		X			X	
Δ82 bp	*pyrE/rph*	intergenic	X		X	X		
+CCTGGC	*rffD*	coding (771/1263 nt)				X		
C→T	*rpoB*	T657I (ACC→ATC)		X				
G→T	*rpoB*	G1189C (GGT→TGT)	X		X	X	X	
+9 bp	*rpoC*	coding (749/4224 nt)						X
G→T	*mrdA*	P481T (CCT→ACT)					X	

^a^ IS = insertion sequence

Notable is the *rpoB* G1189C mutation, occurring in four of the six endpoint strains. Such an occurrence is highly unlikely unless this specific mutation is either highly causal or lays in a hotspot region for mutations, but a more plausible explanation is that it arose in the overnight culture used to inoculate the initial flask of the ALE experiment and was subsequently enriched for. The repeat appearance of the same *rhsE* mutations suggests a similar possibility. To address these issues of genetic reproducibility the smaller scale “reALE” was started with triplicate of the wild-type and triplicate of a ^12^C-glucose pre-evolved strain. Of the three ^12^C pre-evolved reALE endpoint clones selected for sequencing, only one had any new mutations relative to its starting genotype, a 1 base pair deletion in *rpoS*. The absence of the *rpoB* G1189C mutation in the genotypes of the three wild-type reALE endpoints revealed that it had likely occurred in the starting culture of the main ALE (S2 File). Although the same *rhsE* SNPs were observed in both the main and reALE, it was subsequently discovered that these mutations were present in the purportedly-isogenic frozen wild-type stock at roughly 50% frequency. Given that *rhsE* is a pseudogene [13] whose knockout does not alter growth rate (0.73 ± 0.01 hr^-1 13^C-glucose vs. 0.73 ± 0.02 hr^-1 12^C-glucose, biological triplicates), it seems highly likely that these *rhsE* mutations are simply neutral genetic hitchhikers.

Mutations in *pyrE*/*rph* are the most frequently occurring in both ^12^C and ^13^C-glucose evolutions, and this is a well known and repeatedly seen genetic change which is thought to relieve a strain-specific defect in pyrimidine biosynthesis when grown on minimal media [14, 15]. Unique to this ^13^C evolution, however, is the appearance of *pyrE*/*rph* mutations other than the ubiquitous 82 base pair deletion, which has been observed multiple times in evolutions to different environments and by different labs [10, 16]. Although any frameshifting mutation that moves the *rph* stop codon closer to the *pyrE* attenuator loop appears to confer a fitness benefit [10], the 82 base pair deletion has thus far only been observed in evolution experiments because it has a large ease-of-acquisition benefit–it is flanked by two 10 base pair repeats, which allows slipped-strand mispairing (SSM) to occur and preferentially delete the region at a much higher frequency than random SNPs or indels arise [15, 17]. Here, for the first time there is evidence for a possible decrease in the ease-of-acquisition benefit of the 82 base pair deletion, and sequencing of strains during midpoints in the evolution revealed even more atypical *pyrE*/*rph* mutations (S3 Fig and S2 File).

To investigate whether growth on ^13^C-glucose was acting to decrease the frequency of SSM during DNA replication, we tested an engineered strain of *E*. *coli* designed to allow determination of this mutational rate [18]. Added to the chromosome of this strain is a region of the *mod* gene from *H*. *influenzae*, which is a site of naturally occurring phase variation caused by SSM that acts on the tetranucleotide (AGTC) repeats present in the sequence, translationally fused to *lacZ*. LacZ will be produced when this construct is in-frame and colonies will appear blue when streaked on plates with X-gal, but SSM-produced insertions/deletions of the tetranucleotide repeat that cause a frameshift will lead to colonies without LacZ that will appear white on the plates. By growing the strain on ^13^C and ^12^C-glucose and calculating the rate of color switching the rate of SSM can thus be determined. However, despite suggestions to the contrary based on the genotypes of the ^13^C-evolved strains, the rate of SSM is not any different on ^13^C-glucose than it is on ^12^C-glucose (Table 2). Although no difference in SSM frequency at this tetranucleotide length scale doesn’t necessarily mean there is no difference at larger (e.g., 82 base pair) scales, SSM is generally only physiologically relevant at the short scale of simple sequence repeats [19].

**Table 2:** Measurement of slipped-strand mispairing mutation rates.

	Switch frequency ± SD (* 10^−4^)	
	Blue—> White	White—> Blue
^**12**^**C-glucose**	52.1 ± 5.6	31.0 ± 10.5
^**13**^**C-glucose**	54.1 ± 9.2	36.7 ± 4.7
**original study**^a^	55.5 ± 14.6	22.3 ± 4.1

^a^ Reference: [18]

### Glucose Isotopic Preference

**Fig 4:**
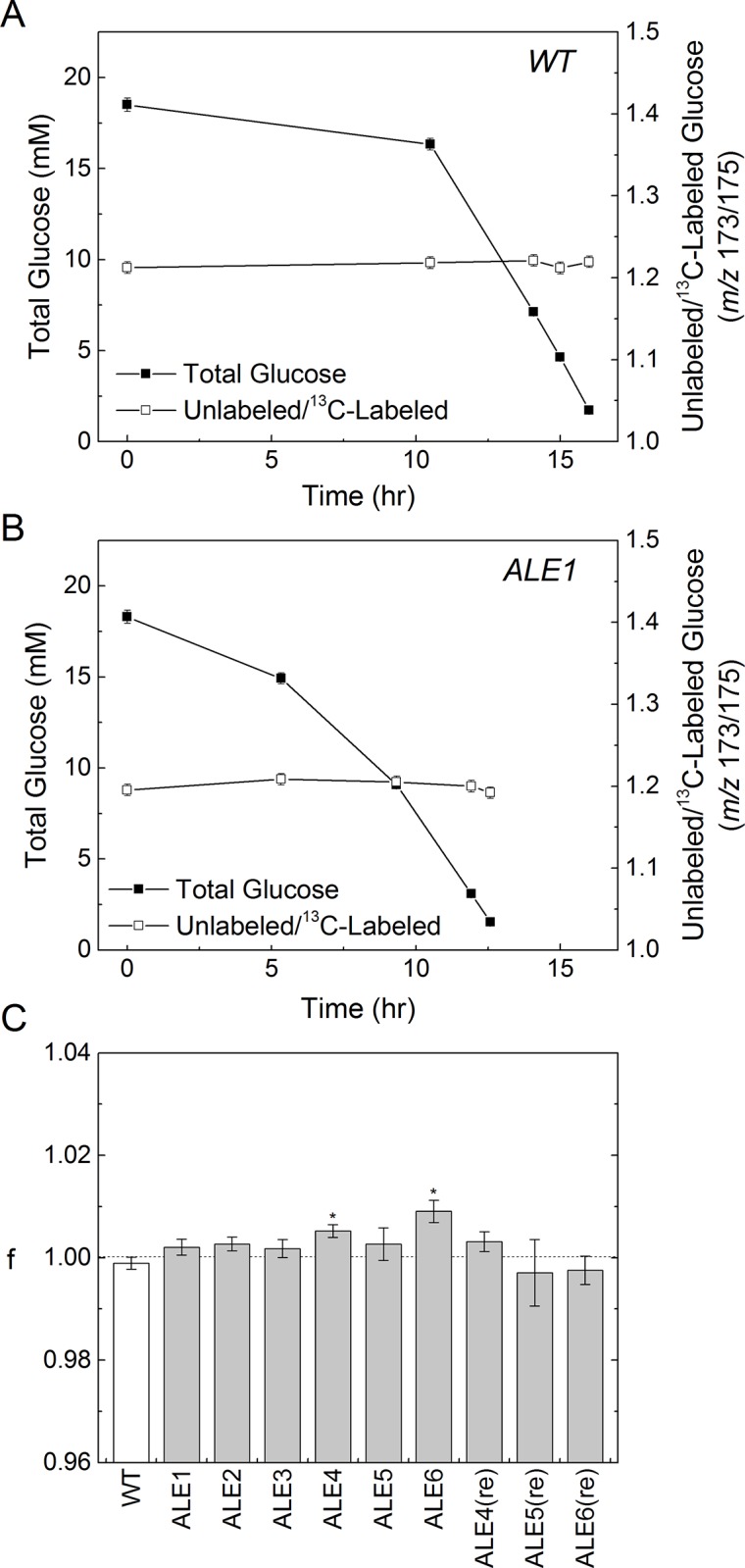
Glucose competition. Results of glucose competition experiments, where the medium contained a mixture of ^12^C-glucose and ^13^C-glucose. Profiles of total glucose concentration and labeling ratio are shown for the WT (A) and one representative ALE strain culture (B). The estimated glucose preference factor “f” reflects the preference for ^12^C-glucose vs. ^13^C-glucose (C). An f-value of 1 reflects no preference, greater than one reflects a preference for ^12^C-glucose, and less than one a preference for ^13^C-glucose.

Although there are as of yet no significant indications that evolution on ^13^C-glucose impacted the cells differently than evolution on ^12^C-glucose, it remains to be seen if these evolved strains exhibit a preference for ^13^C, or similarly if the wild-type prefers ^12^C. The effects of a kinetic isotope effect would manifest in a (perhaps quite slight) difference in uptake rates of glucose based on carbon isotopic composition. This phenomenon was tested for by growing all cultures on a mixture of both ^12^C-glucose and ^13^C-glucose, and monitoring total glucose concentration and the isotopic composition over time (Fig 4 and S4 Fig). The calculated preference factors (f) for all strains are reported in Fig 4C. An f-value greater than 1 indicates preference for unlabeled glucose, while a value less than 1 reflects a preference for ^13^C-glucose. The only statistically significant (*p* < 0.05) preferences were small (less than 1%) preferences for ^12^C-glucose in ALE4 and ALE6. We suspected that these fits were affected by relatively larger GC-MS measurement errors at low glucose concentrations, and upon repeating the experiment with ALE4, ALE5, and ALE 6 we observed no statistically significant preferences (Fig 4C and S5 Fig). Notably, there was no evidence for a preference for ^12^C-glucose in the wild-type (i.e., no kinetic isotope effect), and no evidence for an evolved preference for ^13^C-glucose in the ALE strains. This result held for two strains, WT and ALE1, also tested in chemostat (glucose-limited) cultures. The f-values were 1.003 ± 0.003 and 1.003 ± 0.006, respectively, demonstrating that there is negligible glucose preference across a range of conditions in which different sugar transporters may be expressed [20].

## Conclusion

In this study, six independent *E*. *coli* cultures were evolved for ~1000 generations on uniformly labeled ^13^C-glucose. The resultant strains were then characterized phenotypically and genetically. The results of these analyses, together with comparisons to an evolution study identical except for its use of unlabeled glucose [9], revealed that the heavier carbon does not significantly change any aspect of the adaptive evolution. Regardless of the isotopic composition of the glucose carbon source, populations evolving in parallel take similar trajectories across the fitness landscape. This fitness improvement is enabled by genetic changes that lead to decreased biomass yield, increased glucose and oxygen uptake rates, and increased acetate production rates. Although this trend holds uniformly, these rate and yield changes differ in their magnitude across the evolved strains despite their roughly identical growth rates. Whole genome sequencing on the evolved strains was performed and compared to the sequences of ^12^C-evolved strains. The only notable genetic dissimilarity was the appearance of atypical *pyrE*/*rph* mutations after ^13^C-evolution, despite no apparent isotopically-induced change in slipped-strand mispairing. Nevertheless, this mutational discrepancy is minor–the key genetic regions selectively mutated to optimize growth rate are identical on both ^12^C-glucose and ^13^C-glucose. The metabolic properties of the strains were also probed with a glucose competition experiment, demonstrating that the wild-type exhibits no preference for unlabeled glucose, and the evolved strains have no preference for ^13^C-glucose. This lack of a kinetic isotope effect is yet another indicator of the negligible influence of heavy carbon on the functioning of cells.

Overall, the data presented herein indicate that ^12^C and ^13^C-glucose are interchangeable with respect to *E*. *coli* growth and metabolism, a validation of the assumption that is made ubiquitously in ^13^C-metabolic flux analysis experiments. This assumption is further supported by results of parallel labeling experiments, in which multiple tracers with diverse ^13^C labeling are used and then simultaneously fit to a global flux solution. In an extreme recent example, Crown et. al successfully fit the results of 14 unique parallel tracer experiments in *E*. *coli* [21]. This would not be possible if any of the tracers caused a change in metabolism significant relative to the tight confidence intervals calculated. Thus, based on distinct data from both ^13^C-evolved and wild-type strains, there is strong evidence that no kinetic isotope effect can be detected in ^13^C-MFA studies with the current best available methods. However, careful consideration should be taken before attempting to extend these results to organisms or conditions other than the one studied herein. For example, Wasylenko and Stephanopoulos have demonstrated via mathematical modeling that isotopic effects can vary significantly across different enzymes and organisms, particularly depending on the distribution of metabolic fluxes around certain carbon-carbon bond breaking reactions, and the resulting effect on isotopic labeling can approach the magnitude of GC/MS error [6]. However, in a recent study Millard et. al demonstrated through systems-level modeling that these local effects are muted by metabolic network properties including flux control distribution and bidirectional isotope exchange [22]. The results presented here support the assertion that the kinetic isotope effect is insignificant at the physiological scale, but caution should still be applied when assessing the interchangeability of ^12^C and ^13^C compounds.

## Materials and Methods

### Materials

Media and chemicals were purchased from Sigma-Aldrich (St. Louis, MO). [1,2-^13^C]glucose (99.5 atom% ^13^C), [1,6-^13^C]glucose (99.5% ^13^C), and [U-^13^C]glucose (98% ^13^C) were purchased from Sigma-Aldrich Isotec (St. Louis, MO). Unless otherwise noted in the text, “^12^C-glucose” refers to naturally labeled glucose and “^13^C-glucose” refers to [U-^13^C]glucose. All experiments were performed in M9 minimal medium, which consisted of 1x M9 salts dissolved in distilled water, 2.0 mM MgSO_4_ and 0.1 mM CaCl. Glucose was added as indicated in the text. All solutions were sterilized by filtration.

### Strain and Evolution

The starting strain for evolution was wild-type *E*. *coli* K-12 MG1655 (ATCC 700926). For ALE, cultures were serially propagated (100 μL passage volume) in 15 mL (working volume) flasks of M9 minimal medium with 2 g/L ^13^C-glucose, kept at 37°C and well-mixed for full aeration. An automated system passed the cultures to fresh flasks once they had reached an OD_600_ of 0.3 (Tecan Sunrise plate reader, equivalent to an OD_600_ of ~1 on a traditional spectrophotometer with a 1 cm path length), a point at which nutrients were still in excess and exponential growth had not started to taper off (confirmed with growth curves and HPLC measurements). Four OD_600_ measurements were taken from each flask, and the slope of ln(OD_600_) vs. time determined the culture growth rates. A cubic interpolating spline constrained to be monotonically increasing was fit to these growth rates to obtain the fitness trajectory curves.

### Whole genome sequencing

Colonies were isolated from evolved populations by streaking on LB agar plates. Genomic DNA was extracted using Promega’s Wizard DNA Purification Kit. The quality of DNA was assessed with UV absorbance ratios using a NanoDrop spectrophotometer. DNA was quantified using a Qubit dsDNA High Sensitivity assay. Paired-end resequencing libraries were generated using a Nextera XT kit from Illumina with 1 ng of total input DNA. Sequences were obtained using an Illumina Miseq with a PE500v2 kit. The breseq pipeline [23] version 0.23 with bowtie2 [24] was used to map sequencing reads and identify mutations relative to the *E*. *coli* K12 MG1655 genome (NCBI accession NC_000913.2). All samples had an average mapped coverage of at least 90x.

### Slipped-strand mispairing frequency measurement

*E*. *coli* strain MV759 [18] was streaked onto an M9 glucose agar plate with X-gal. One white colony and one blue colony were selected and used to inoculate flasks of both ^12^C and ^13^C-glucose M9 medium. After growing up, a volume of these cultures was plated onto ^12^C or ^13^C-glucose M9 agar plates with X-gal, and the number of cells of each color was counted. This process was repeated independently three times, and an average of ~1200 cells were counted on each plate. The SSM-induced color switching frequency was calculated by dividing the fraction of cells that had switched color by the generations of growth that had occurred.

### Glucose competition experiments

Glucose competition experiments were performed to determine the preference of the various *E*. *coli* strains for ^12^C vs. ^13^C-glucose. Cells were first pre-cultured overnight in medium containing 2 g/L ^12^C-glucose. Next, 50 μL of the overnight pre-culture was used to inoculate 10 mL of M9 medium containing approximately equal amounts (2 g/L) of ^12^C-glucose and ^13^C-glucose. Total glucose concentration was measured over time, as well as ^13^C-labeling of glucose remaining in the medium. Based on this data, a preference factor (f) was calculated, defined as follows:

$$\frac{uptake\;rate\;of\;unlabeled\;glucose}{uptake\;rate\;of\;labeled\;glucose}=\text{f}\times \frac{fraction\;of\;unlabeled\;glucose\;in\;medium}{fraction\;of\;labeled\;glucose\;in\;medium}$$

An f-value greater than one indicates a preference for ^12^C-glucose, while a value less than one reflects a preference for ^13^C-glucose. To determine the f-value, the following expression was used (see S4 File for derivation):

$$ln\left(\frac{x_{unlabeled}\left(t\right)\;*\;gluc\left(t\right)}{x_{unlabeled}\left(t=0\right)\;*\;gluc\left(t=0\right)}\right)=f\;*\;ln(\frac{x_{labeled}(t)\;*\;gluc(t)}{x_{labeled}(t=0)\;*\;gluc(t=0)})$$

Where, *gluc(t)* is the measured glucose concentration over time, x_unlabeled_(t) is the fraction of ^12^C-glucose in the medium over time, and x_labeled_(t) is the fraction of ^13^C-glucose in the medium over time. The f-parameter and its error estimate were obtained with the above equation by one-parameter linear least-squares regression. To reduce sensitivity to measurement error in the initial values (t = 0), the average fractional labeling of the first two samples was used as x_labeled_(t = 0) and x_unlabeled_(t = 0).

For the competition experiments in chemostat mode, M9 feed medium with approximately 3 mM each of ^12^C-glucose and ^13^C-glucose was prepared. The working cell culture volume was 15 mL, and the feed rate was 3 mL/hr, resulting in a dilution rate of 0.2 hr^-1^. Media samples were taken after 20 hours, well after steady state was established. Error estimates of the f-value were obtained by propagating the typical GC/MS error of 0.3%.

### Analytical methods

Cell growth in glucose competition experiments was monitored by measuring the optical density at 600nm (OD_600_) using a spectrophotometer (Eppendorf BioPhotometer). The OD_600_ values were converted to cell dry weight concentrations using a pre-determined OD_600_-dry cell weight relationship for *E*. *coli* (1.0 OD_600_ = 0.32 g_DW_/L). Glucose concentration was measured with a YSI 2700 biochemistry analyzer (YSI, Yellow Springs, OH). Acetate concentration was measured by HPLC [25]. No compounds other than glucose and acetate were detected in the cultures.

### Determination of yields and biomass specific rates

Yield of biomass on glucose was determined as the slope of least-squares regression of biomass concentration versus glucose concentration [26]. Acetate yield was determined based on the initial and final glucose and acetate concentrations in batch cultures. Specific growth rate (GR, h^-1^) was determined as the slope of least-squares regression of ln(OD_600_) versus time during the exponential growth phase, typically for OD_600_ values between 0.01 and 0.7. Growth rates as presented in Fig 2A and S2 Fig represent “physiologically adapted” rates [10], i.e. cultures were kept in exponential phase growth for three flasks so that their growth rates stabilized and were no longer decreased due to recently coming out of stationary phase [27]. This was to allow for comparison with the fitness trajectories, which inherently represent physiologically adapted rates. For calculations of compound uptake/production rates, the non-physiologically adapted (i.e. from the first post-overnight culture growth flask) growth rates were used, to allow for proper comparison with other studies (values given in S1 File). Specific glucose uptake (GUR, mmol/g_DW_/h) was determined as the ratio of growth rate to biomass yield. Specific acetate production rate (APR, mmol/g_DW_/h) was determined as the product of specific glucose uptake rate and acetate yield. Specific oxygen uptake (OUR, mmol/g_DW_/h) was determined based on electron balance as described previously, assuming a degree of reduction of 4.35 and a molecular weight of 0.0255 g_DW_/mmol-C for dry biomass [28]:

$$OUR\;\left(\frac{mmol}{g_{DW}*h}\right)=\frac{24*GUR-8*APR-\frac{4.35}{0.0255}*GR}{4}$$

### Gas chromatography mass spectrometry

GC-MS analysis was performed on an Agilent 7890B GC system equipped with a DB-5MS capillary column (30 m, 0.25 mm i.d., 0.25 μm-phase thickness; Agilent J&W Scientific), connected to an Agilent 5977A Mass Spectrometer operating under ionization by electron impact (EI) at 70 eV [29]. Labeling of glucose was determined by GC-MS analysis of the aldonitrile pentapropionate derivative of glucose [30]. The fragments at *m/*z 173 and *m/*z 370 were analyzed, which contain the last two and first five carbon atoms of glucose, respectively. Mass isotopomer distributions were obtained by integration and corrected for natural isotope abundances [31].

## Supporting Information

S1 Fig Fitness trajectories for reALE. Fitness trajectories for a smaller scale “reALE” on ^13^C-glucose, starting from either the wild-type (reALE 1–3) or a strain that was pre-evolved on unlabeled glucose (reALE 4–6).
(PNG)

S2 Fig reALE growth rates. Growth rates of endpoint strains for the small-scale reALE. Of the three pre-evolved endpoint colonies, only one had any new mutations (a 1 base pair *rpoS* deletion in reALE5).
(PNG)

S3 Fig Individual trajectories with sequencing points. Individual fitness trajectories for the main ALE, with circled points representing where colonies were isolated from the populations and sequenced (full sequencing results in S2 File).
(PNG)

S4 Fig Full glucose competition experiments. Glucose consumption and isotopic labeling data for all tested strains.
(TIF)

S5 Fig Repeated glucose competition experiments. Glucose consumption and isotopic labeling data for the re-analysis of ALE4, ALE5, and ALE6.
(TIF)

S1 File Physiological data. (XLSX)

S2 File Sequencing data. (XLSX)

S3 File Glucose competition data. (XLSX)

S4 File Glucose preference derivation. (DOCX)
