# Supplementary figures and images for: Evolution of E. coli on [U-13C]Glucose Reveals a Negligible Isotopic Influence on Metabolism and Physiology
Source: PLoS One. 2016 Mar 10;11(3):e0151130. doi: 10.1371/journal.pone.0151130 (PMC4786092; doi:10.1371/journal.pone.0151130)

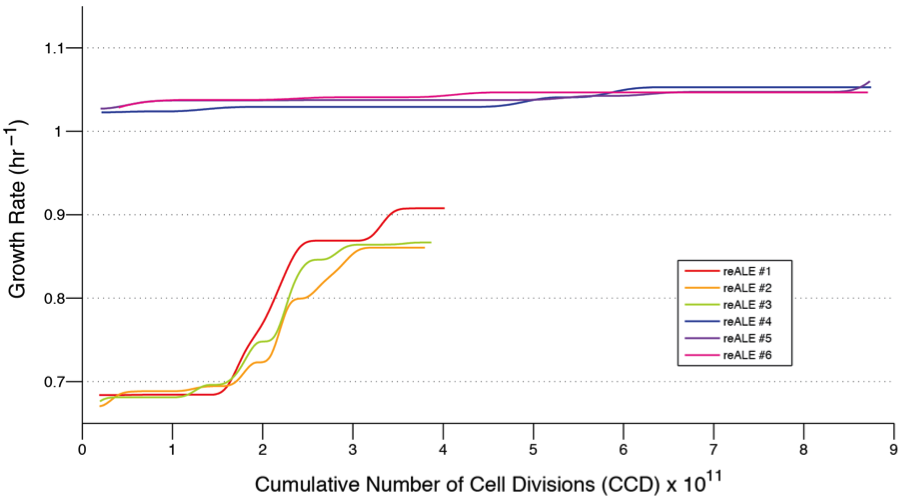

Supplement: S1 Fig — Fitness trajectories for a smaller scale “reALE” on 13C-glucose, starting from either the wild-type (reALE 1–3) or a strain that was pre-evolved on unlabeled glucose (reALE 4–6). (PNG) [file pone.0151130.s001.png]

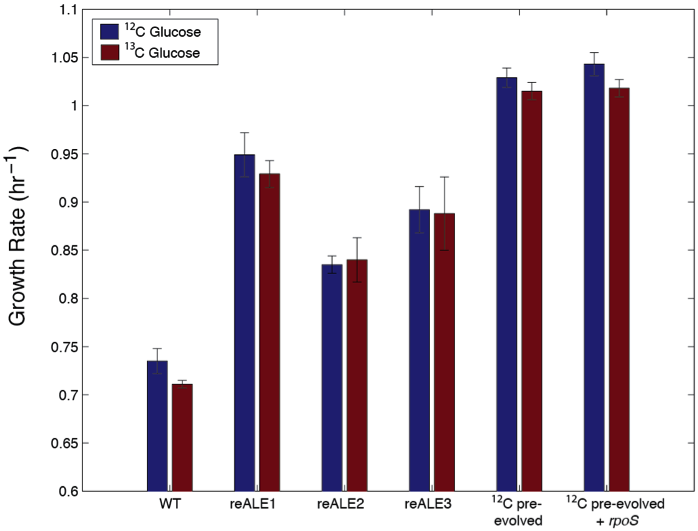

Supplement: S2 Fig — Growth rates of endpoint strains for the small-scale reALE. Of the three pre-evolved endpoint colonies, only one had any new mutations (a 1 base pair rpoS deletion in reALE5). (PNG) [file pone.0151130.s002.png]

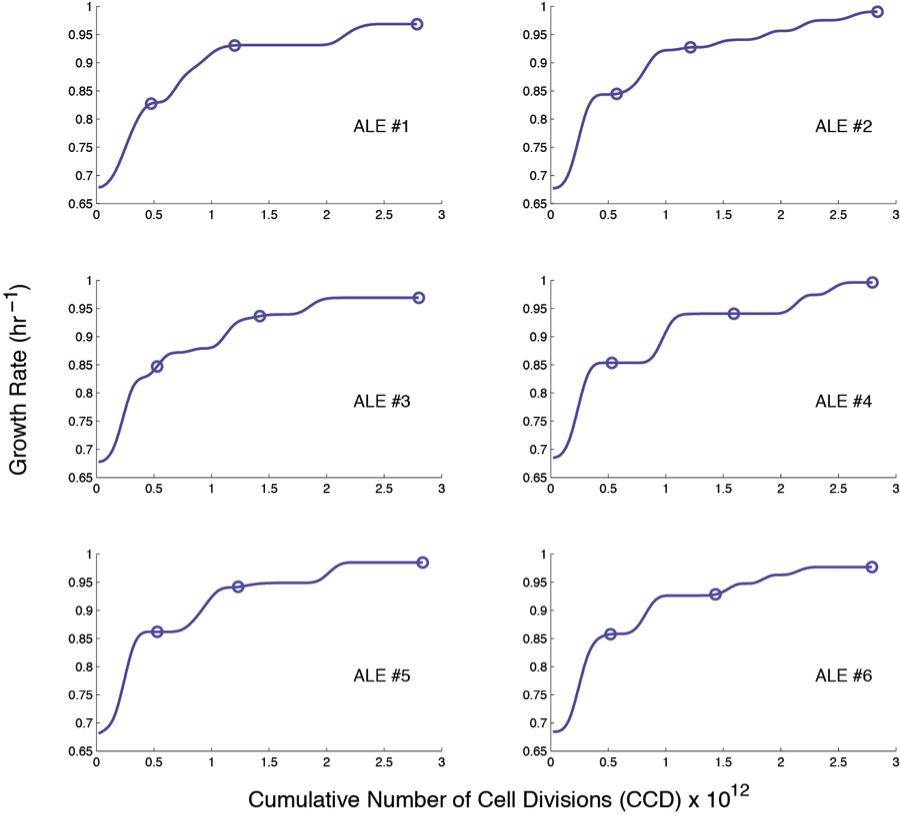

Supplement: S3 Fig — Individual fitness trajectories for the main ALE, with circled points representing where colonies were isolated from the populations and sequenced (full sequencing results in S2 File). (PNG) [file pone.0151130.s003.png]
