# Supplementary material for: Evolution of E. coli on [U-13C]Glucose Reveals a Negligible Isotopic Influence on Metabolism and Physiology
Source: PLoS One. 2016 Mar 10;11(3):e0151130. doi: 10.1371/journal.pone.0151130 (PMC4786092; doi:10.1371/journal.pone.0151130)
Supplement: S4 File — (DOCX) [file pone.0151130.s009.docx]

**Derivation of equation to calculate glucose preference factor from glucose competition experiments.**

The preference factor (f) is defined by the following equation:

$\frac{uptake of unlabeled glucose}{uptake of labeled glucose}=f*\frac{fraction of unlabeled glucose in medium}{fraction of labeled glucose in medium}$ (1)

Thus, an f-value greater than one indicates a preference for ^12^C-glucose, while a value less than one reflects a preference for ^13^C-glucose.

Nomenclature for the equations below:

U = concentration of unlabeled glucose in the medium (mM)

U0 = initial concentration of unlabeled glucose in the medium (mM)

L = concentration of labeled glucose in the medium (mM)

L = initial concentration of labeled glucose in the medium (mM)

G = total concentration of glucose in the medium (mM)

G0 = initial concentration of glucose in the medium (mM)

xU = fraction of unlabeled glucose in the medium (-)

xU0 = initial fraction of unlabeled glucose in the medium (-)

xL = fraction of labeled glucose in the medium (-)

xL0 = initial fraction of labeled glucose in the medium (-)

Equation 1 can be written as:

$\frac{dU/dt}{dL/dt}=f*\frac{xU}{xL}=f*\frac{U}{L}$ (2)

$\frac{dU}{dL}=f*\frac{U}{L}$ (3)

Integration of Eq (3) with the boundary condition at t=0: U(t=0) = U0, L(t=0)= L0 yields:

$ln(\frac{U}{U0})=f*ln(\frac{L}{L0})$ (4)

Substituting U = G*xU, U0 = G0*xU0, L = G*xL, L0 = G0*xL0, produces the final equation:

$ln(\frac{xU*G}{xU0*G0})=f*ln(\frac{xL*G}{xL0*G0})$ (5)

Or, as written out in the paper:

$ln\left( \frac{x_{unlabeled}\left( t \right)*gluc\left( t \right)}{x_{unlabeled}\left( t=0 \right)*gluc\left( t=0 \right)} \right)=f*ln(\frac{x_{labeled}(t)*gluc(t)}{x_{labeled}(t=0)*gluc(t=0)})$ (6)
